# Improved Process to Obtain Nanofibrillated Cellulose (CNF) Reinforced Starch Films with Upgraded Mechanical Properties and Barrier Character

**DOI:** 10.3390/polym12051071

**Published:** 2020-05-07

**Authors:** Luis Angel Granda, Helena Oliver-Ortega, Maria José Fabra, Quim Tarrés, Maria Àngels Pèlach, José Maria Lagarón, José Alberto Méndez

**Affiliations:** 1 LEPAMAP Group, Department of Chemical Engineering, University of Girona EPS, PI, Maria Aurèlia Capmany 61, 17003 Girona, Spain; lgranda@leitat.org (L.A.G.); helena.oliver@udg.edu (H.O.-O.); angels.pelach@udg.edu (M.À.P.); 2 Instituto de Agroquímica y Tecnología de Alimentos (IATA-CSIC), Agustín Escardino 7, Paterna, 46980 Valencia, Spain; mjfabra@iata.csic.es; 3 Novel Materials and Nanotechnology Group, IATA, CSIC. Av. Agustín Escardino 7, Paterna, 46980 Valencia, Spain; lagaron@iata.csic.es

**Keywords:** biodegradable nanocomposites, starch, nanofibrillated cellulose, barrier properties

## Abstract

Nowadays, the interest on nanofibrillated cellulose (CNF) has increased owing to its sustainability and its capacity to improve mechanical and barrier properties of polymeric films. Moreover, this filler shows some drawbacks related with its high capacity to form aggregates, hindering its dispersion in the matrix. In this work, an improved procedure to optimize the dispersability of CNF in a thermoplastic starch was put forward. On the one hand, CNF needs a hydrophilic dispersant to be included in the matrix, and on the other, starch needs a hydrophilic plasticizer to obtain a thermoformable material. Glycerol was used to fulfil both targets at once. CNF was predispersed in the plasticizer before nanofibrillation and later on was included into starch, obtaining thin films. The tensile strength of these CNF–starch composite films was 60% higher than the plain thermoplastic starch at a very low 0.36% *w*/*w* percentage of CNF. The films showed a noticeable correlation between water uptake, and temperature and humidity. Regarding permeability, a ca. 55% oxygen and water vapor permeability drop was found by nanofiller loading. The hydrolytic susceptibility of the composite was confirmed, being similar to that of the thermoplastic starch.

## 1. Introduction

Nowadays, the interest in natural resources to produce natural, renewable, and environmentally friendlier materials has increased. This social trend has promoted the enhancement of research related to the study of nanoscale products derived from cellulose nanofibers. Among these materials, nanofibrillated cellulose (CNF), together with cellulose nanocrystals (CNCs), are among the most relevant. Aside from the abovementioned environmental criteria, CNF’s intrinsic characteristics—high water uptake capacity, high strength, insolubility in common solvents, and low gas permeability rates—increase the interest in such reinforcements. These properties have promoted CNF for its use in many applications such as biomedical [1], papermaking [2,3], production of nanopaper [4,5], foods and paintings modifier [6,7], aerogel production [8,9,10], and as a reinforcing agent for polymer matrices [11,12]. Moreover, hybrid systems based on CNF together with other inorganic compounds, such as graphene [13,14], calcium carbonate [15], or even magnetic particles [16], have been effectively synthesized to produce systems with fire-retardant capacity or for packaging as well as construction purposes.

CNF is obtained by the mechanical and high-pressure disintegration of biomass by obtaining fibrous materials with very high aspect ratios [17]. The common dimensions of nanofibrillated cellulose, reported in literature, show diameters lower than 100 nm and lengths of several micrometers [18]. It is worth mentioning that the literature indistinctly used the terms nanofibrillated cellulose (CNF) and microfibrillated cellulose (MFC).

The polymeric matrices used in CNF reinforced composites are those capable of chemically interacting with cellulose at the interphase surface, with the most relevant being poly(vinyl alcohol) (PVA) [19], thermoplastic starch (TPS) [20], and chitosan (CHI) [21]. Using these matrices, together with the high aspect ratio as well as the high specific surface of CNF, an effective interaction is reached. This interaction is based on the establishment of hydrogen bonding between the –OH groups of the polymer chain and those of the nanofibers [22].

Moreover, some hydrophobic matrices can be reinforced with CNF, but a compatibilization process of the nanofibers, prior its compounding with the matrix, must be performed to induce its dispersibility. This is the case of the chemical surface functionalization of CNF to improve its compatibility with non-polar polymers. In this sense, chemical modifications, such as post-sulfonation, acetylation [23], acetylation [18], and polymer grafting [18] have been reported.

In addition to the chemical compatibility of the phases, the processing techniques to obtain CNF-based composites also play an important role related to the difficulty to disperse the nanofibers into the polymer matrices. One of the most often used methods to produce films of polysaccharides reinforced with cellulose nanofibers is solvent casting. Savadekar and Mhaske [24] obtained a composite film based on starch as a polymer matrix and CNF derived from short stable cotton fibres as reinforcement. They found that the incorporation of 0.4% *w*/*w*, regarding starch content, increased the tensile strength by 46%. They also reported that the composite properties’ improvements depended on different parameters: content of CNF, degree of dispersion of the nanofibers in the polymer matrix, and matrix–fiber interactions. In this sense, the dispersion of CNF in the polymer was one of the most influent parameters. Other authors reached similar conclusions, where the incorporation of an increasing percentages of CNF led to the formation of aggregates that limited the improvement of mechanical strength as well as reduced the capacity of deformation [12]. Karimi et al. prepared composite starch biofilms reinforced with CNF obtained from retted kenaf fibres. The solvent casting of composites reinforced with 0.5% *w*/*w* of nanofibers led to materials with improvements of mechanical strength by 3.4% and 35%, depending on the processing procedure of each component of the composite. Moreover, the elongation at break of these films showed a slight decrease in the range of 1.2% to 19%.

Multifunctional alcohols, such as glycerol or sorbitol, are regarded as potential starch plasticizers owing to the H-bond interactions established between the –OH functionality of cellulose and that of the alcohol. This plastifying process often leads to a material with poor mechanical properties, and if the percentage of plasticization is quite high, other properties, such as barrier capacity, are also harmed [25]. One common way to improve such barrier properties is including nanofibers into the matrix based on the incorporation of a highly diluted aqueous suspension of CNF to the native material and the plasticizers, in order to allow water evaporation and subsequent film formation. This process can result in CNF–CNF interactions (hydrogen bonding) that produce aggregates, which decreases the effective transmission of stresses from the matrix to the nanofibers, decreasing the tensile strength and elongation at break of the composites [12,26].

Another process to produce CNF reinforced starch composites is based on an initial blending of the matrix and a masterbatch of CNF (predispersed) and a later plasticization of the final material, extrusion, and a subsequent film transformation using heat compressing [26]. Hietala et al. applied this methodology to transform starch–CNF-based composites into film-shaped materials with a percentage of CNF, derived from softwood flour, in the range from 5% to 20% *w*/*w*. In this case, 20% *w*/*w* of CNF was needed to improve tensile strength by 100%, compared with plain TPS. Again, an important 90% drop in elongation at break was observed. This result confirms a high correlation between the mechanical properties of the composite and the processing methodologies.

In this work, an evolution of a conventional processing methodology was used to prepare thermoplastic starch films reinforced with nanofibrillated cellulose (CNF). Bleached eucalyptus pulp (BEP) was pre-treated using a 2,2,6,6-tetramethylpiperidine 1-oxyl (TEMPO)-assisted oxidation in an aqueous medium to promote the formation of carboxylic functionality on carbon 6 [27] of the cellulose structure. Once the fibers were oxidized, the solvent was removed by filtration without reaching a dry state. Later on, the fibers were dispersed in glycerol, which is the same dispersant that will be used as a plasticizer for starch. The BEP-glycerol dispersion was homogenized to allow fibrillation, giving rise to a diluted CNF-glycerol gel-like dispersion that was added to starch, this being the key step of the proposed process evolution. An improvement in CNF dispersion was expected after homogenizing the suspension in a higher viscous media that can avoid aggregation improving tensile strength and barrier character. Starch was plasticized at the same time as CNF addition proceeded in an internal mixer. Thereafter, composites were transformed into film-shaped specimens with a thickness lower than 250 μm. Mechanical testing, water uptake behavior under different environmental conditions, hydrolytic susceptibility evaluation, and barrier character against water vapour and oxygen were performed to determine the competitiveness of the material as food contact films.

## 2. Materials and Methods

### 2.1. Materials

Bleached eucalyptus pulp (BEP), supplied by Emcee, Navia, Spain, was used as cellulosic resource to produce nanofibrillated cellulose (CNF). 2,2,6,6-tetramethylpiperidine 1-oxyl (TEMPO) (98% purity, Sigma Aldrich, Madrid, Spain) used as catalyst, sodium bromide (NaBr, ≥99% purity), sodium hypochlorite (NaClO, 5% *w*/*w* of chlorine), and sodium hydroxide (NaOH, 98% purity), all supplied by Scharlau, Barcelona, Spain, were the reactants used in the TEMPO-mediated oxidation.

Potato starch (food grade, applied for food market) was provided by Avebe, Veendam, The Netherlands and used, without any prior purification, as biodegradable polymer matrix. Glycerol (95% purity) was provided by Scharlau, Barcelona, Spain and added as a plasticizer. 

α-amylase (Aquazym 480L, declared activity 480 KNU-B/g) was kindly provided by Novozymes, Baggsværd, Denmark and used as received to perform the enzymatic hydrolytic study.

### 2.2. Methods

*Pre-treatment of BEP fibers:* BEP fibers were pre-treated using an oxidation process catalyzed by TEMPO [27]. In a typical experiment, 15 g of dry BEP was dispersed in distilled water at a concentration of 1% *w*/*w*, containing a catalytic composition of TEMPO (0.016 g per g of fibers) and NaBr (0.1 g per g of fibers). The viscous dispersion was stirred for 15 min at room temperature. After time elapsed, an aqueous solution of NaClO (15% *w*/*w*) was added dropwise to the slurry. NaClO was calculated to add 5 mmol, 10 mmol, and 15 mmol, in independent experiments, per gram of BEP fibres. The pH was kept at 10 by the addition of a 0.5M NaOH aqueous solution. The oxidation was considered finished when the pH was stabilized a pH 10 without addition of NaOH. The oxidized fibers were filtered and washed three times with distilled water.

*Nanofibrillation of BEP fibers (CNF*_BEP_*)*: The dry mass of the filtered oxidized BEP fibers was measured by drying in an oven at 105 °C to constant weight. Once the concentration was known, the dispersion was concentrated without totally removing the water and added to glycerol to obtain a value of concentration of 1.0% at room temperature using a magnetic stirrer. The diluted dispersion was passed five times through a homogenizer supplied by GEA Mechanical Equipment, Bolonia, Italy. The final pressure of the homogenizer was higher than 600 bar. The gel obtained by this process was ready to be added to the polymer matrix.

*Processing of the starch–CNF*_BEP_*composite*: Native starch, *CNF*_BEP_ dispersed in glycerol, and water was hand-mixed in a linear low-density polyethylene (LLDPE) bag to reach a homogeneous sticky slurry. The bag was closed and kept at room temperature for 24 h. Thereafter, the blend was introduced in an internal mixer (Brabender Plastograph^®^, Duisburg, Germany). In a typical experiment, 50 g of blend was mixed at 110 °C for 12 min at a mixing speed of 100 rpm. The processed composite was stored at room temperature inside closed LLDPE bags until film transformation.

*Thin film transformation*: 13 g of starch–*CNF*_BEP_ composite was thermoformed using a hydraulic LabEcon series press (Fontijne Grotnes, Vlaardingen, The Netherlands) to obtain thin films (approximately 250 μm). The material was placed between the two warm plates without physical contact for 5 min at 135 °C. After time elapsed, the material was compressed by the plates with a force of 100 kN. This pressure was maintained for 10 min, and then increased up to 150 kN for 5 more minutes. The thermoforming process was finished, and the plates were cooled to 60 °C. The films were removed and stored in LLDPE bags until characterization.

*Microscopic study*: Transmission electron microscopy (TEM) was carried out with a EM910 microscope (ZEISS, Oberkochen, Germany) operating at 120 kV. Specimens for inspection by TEM were prepared by slowly evaporating one drop of the LCNF gel, at reduced pressure on a 400 mesh copper grid, coated by a carbon-supported film.

*Mechanical testing*: Starch–*CNF*_BEP_ composite films were cut into 180 × 15 × 0.250 mm (length × width × thickness) specimens, according to ASTM D 882-02 standard specifications. Prior to testing, the specimens were stored for at least 40 h in a climatic chamber model 3000, Dycometal, Viladecans, Spain at temperature (T) of 23 ± 1 °C and 7 ± 1 °C, in independent experiments, and 50% relative humidity (RH). The specimens were tested under tensile stress, without extensometer, in a Hounsfield Universal Testing Machine (Shakopee, MN, USA) equipped with a load cell of 2.5 kN.

*Water uptake capacity* (W.U.): Tensile test specimens were used to determine the water uptake behavior of the composite films. The specimens were dried before testing at 50 °C to constant weight. A minimum of three samples were stored in a climatic chamber (Dycometal) applying three different conditions, in independent assays: (a) 23 ± 1 °C/50% RH, (b) 23 ± 1 °C/75% RH, and (c) 7 ± 1 °C/50% RH. The samples’ weight was measured periodically until a stabilization state was reached, to determine the water uptake behavior. The water uptake content (WU%) was calculated using Equation (1):

(1) $$\mathrm{WU}\%=\frac{w_{t}-w_{0}}{w_{0}}\times 100$$

where w_0_ and w_t_ are the initial (dry) weight and after t time in the climate chamber, respectively.

*Hydrolytic susceptibility in enzymatic media*: Fragments of samples were soaked in an aqueous solution (20 mL, 6·× 10^−2^ KNU/L) of α-amylases and stored at 50 °C according to the provider’s instructions. The samples were removed at different time periods to complete a four-day exposition time. The hydrolysis was stopped by adding one drop of an aqueous solution on acetic acid (2M). The weight loss was calculated using Equation (2):

(2) $$\mathrm{Weight}\;\mathrm{loss}\%=\frac{w_{0}-w_{t}}{w_{0}}\times 100$$

where w_0_ and w_t_ are initial (dry) weight and after immersion time t, respectively.

*Determination of water vapour permeability*: Direct permeability to water vapour was determined from the slope of weight loss versus time curves at 24 °C [28]. The films were placed between an aluminum plate (open O-ring) and a deposit for the permeant (distilled water), designed as a permeability cell. A Viton rubber O-ring was placed between the film and the aluminum plate of the cell to enhance tightness. The cells were placed inside a desiccator at 0% RH, and the water weight loss through a film area of 0.001 m^2^ was monitored and plotted against time. To estimate the permeability values of the films, only the linear part of the weight loss data was used to ensure sample steady-state conditions. Cells with aluminum films were used as control specimens to determine solvent loss through the sealing. Water weight loss was calculated as the total cell weight loss minus the loss through the sealing. Every experiment was performed in duplicate.

*Determination of oxygen permeation*: The oxygen permeability coefficient was derived from the oxygen transmission rate (OTR) measurements recorded, in duplicate, using an oxygen permeation analyzer M8001 (Systech Illinois, P2RJ+4W Thame, UK) set at 40% RH and 23 °C. The samples were previously purged with nitrogen to degas the materials and condition them at the selected environmental conditions, before exposure to an oxygen flow of 10 mL·min^−1^. The tested sample area was 5 cm^2^ for all materials to minimize the effect of potential thickness variations and defects. To obtain the oxygen permeability, the OTR was corrected with film thickness and the gas partial pressure used.

*Statistical study*: The statistical analysis of the mechanical properties of the composite films and that of the gas permeation were performed by an analysis of variance (ANOVA). In all statistical analyses, *p* < 0.05 was considered as statistically different.

## 3. Results and Discussion

### 3.1. Processing of the Composite Films

One of the main problems in the preparation of composite materials reinforced with nano-sized reinforcements is achieving a correct dispersion of the reinforcements in the polymer matrices. The most often used methodology for the preparation of nanofiber reinforced films is based on its dispersion in a matrix solution, and then removing the solvent by evaporation (solvent casting). Using this method, the production of poly(vinyl alcohol) (PVA) and thermoplastic starch (TPS) composite films is possible. Nonetheless, the mechanical properties of the composite are strongly linked to the degree of dispersion of the nano-reinforcement in the polymer matrix.

Additionally, another problem, derived from the partial solubility of starch in water, was observed, which is dependent on the intrinsic chemical composition: source and amylose/amylopectin composition ratio.

In this work, a combined method was performed to produce starch nanocomposites through the incorporation of the reinforcement already pre-dispersed in the plasticizer (glycerol).

**Figure 1:**
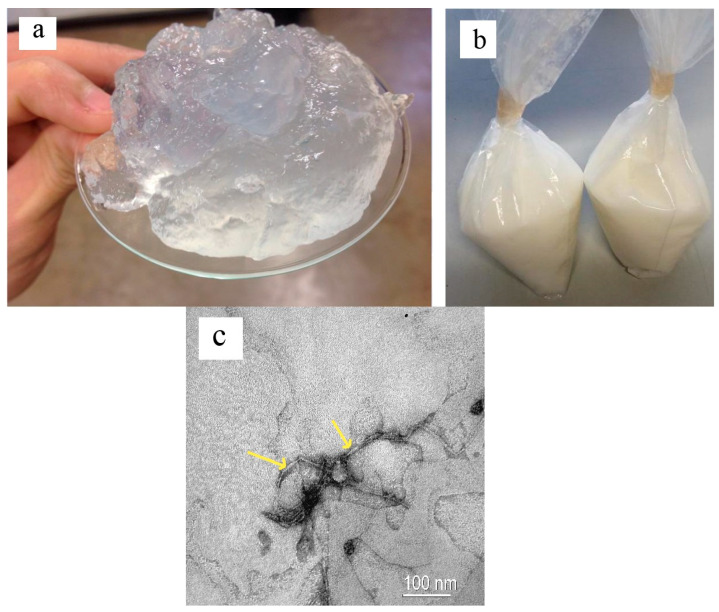
(**a**) Gel based on nanofibrillated cellulose (CNF)_BEP_ dispersed in glycerol (gly) (1% *w*/*w* of concentration); (**b**) mixture of native starch + *CNF*_BEP_ dispersion in glycerol + distilled water inside the linear low-density polyethylene (LLDPE) bags; (**c**) transmission electron microscopy (TEM) picture of the gly-*CNF*_BEP_ gel. BEP, bleached eucalyptus pulp.

Once the oxidation pre-treatment of *CNF*_BEP_ was finished, the resulting oxidized fibers were washed and filtered, without completely drying them, to avoid *CNF*_BEP_ agglomeration. Later, the *CNF*_BEP_ were dispersed in glycerol to obtain a concentration by 1.0% *w*/*w*. The processing of the gly- *CNF*_BEP_ dispersion through the homogenizer was used to reach two goals: (1) performance of the nanofibrillation process to obtain the characteristic gel-like concentration, and (2) to induce an optimal dispersion of the *CNF*_BEP_ in the gel through the application of high pressure. Figure 1a shows the gel-like structure of the glycerol-*CNF*_BEP_ gel after homogenization by applying 600 bars of pressure. The glycerol-*CNF*_BEP_ gel was added to native starch and distilled water. The blend was mixed inside an LLDPE bag and subsequently kept at room temperature for 24 h (Figure 1b) before the internal mixing. Figure 1c shows TEM pictures of the glycerol-*CNF*_BEP_ gel, where it is possible to observe the nanometric dimensions of the fibres, lower than 20 nm in thickness (labelled with yellow arrows), as well as the good individualisation characteristic induced by the homogenization procedure.

**Figure 2:**
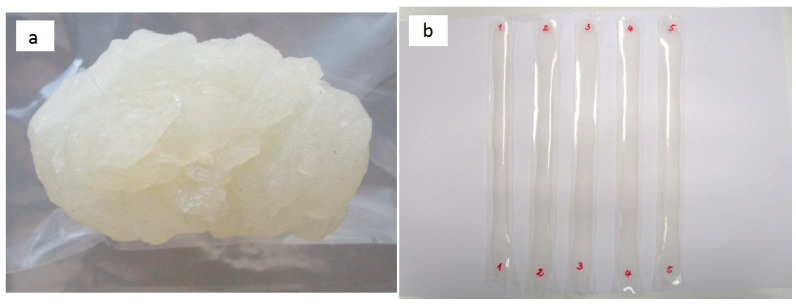
(**a**) Composite 10ST40 after processing in the internal mixer; (**b**) 10ST40 composite film specimens for tensile characterization.

The processing of the starch, together with the glycerol-*CNF*_BEP_ gel and water, was carried out at a moderate temperature and mechanical stresses in the internal mixer, producing the amorphous/viscous material shown in Figure 2a. Table 1 summarizes all formulations of thermoplastic starch reinforced with *CNF*_BEP_ fibers derived from BEP. The percentage of *CNF*_BEP_ refers to the composition of nanofibrillated cellulose in the feed.

All materials were transformed into thin completely transparent films (Figure 2b), with thickness comprised between 200 to 250 μm.

### 3.2. Water Uptake Behaviour (W.U.)

**Figure 3:**
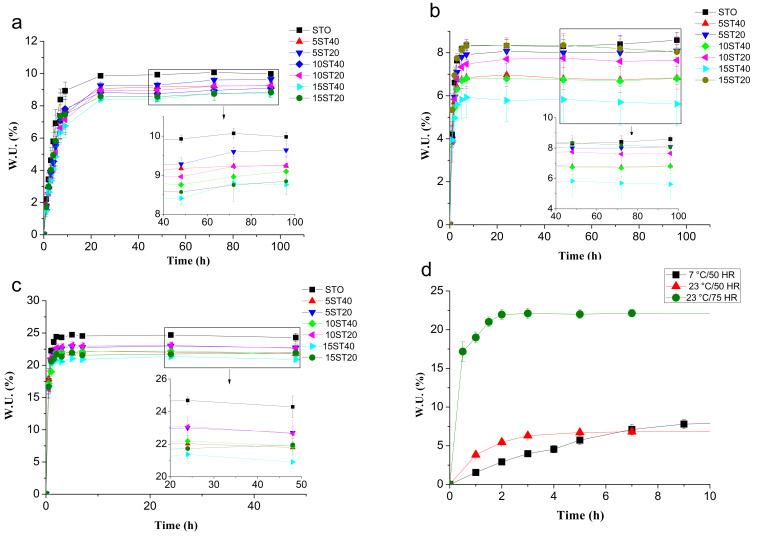
Water uptake profiles of starch composite films reinforced with *CNF*_BEP_. Conditions: (**a**) 7 °C/50% relative humidity (RH), (**b**) 23 °C/50% RH, and (**c**) 23 °C/75% RH. (**d**) Comparative water uptake profile of formulation 10ST40 under different conditions. W.U., water uptake.

The materials were submitted to different environmental conditions to determine their capacity for water swelling and its dependence with temperature and humidity. The results of the study are shown in Figure 3. The first observation that can be stated is the increase in the rate of water uptake when temperature increases, assuming that the interaction between starch and the cellulose nanofibers reduces the water diffusion capacity through the material. This increase in the rate of water absorption can be observed during the first 3 h of the experiment and is shown in Figure 3d, where the profiles of W.U. versus time of the formulation 10ST40, by applying different environmental conditions, are plotted. Comparing the trend 7 °C/50% RH with 23 °C/50% RH, it is easy to observe that the initial slope of the first plot is lower than that of the second. From a quantitative point of view, the increase of temperature is associated with a water diffusion coefficient enhancement. The numerical determination of this coefficient could not be obtained because of the fast water uptake capacity of the films, which did not allow the acquisition of enough data to fulfil the requirements of the calculation by the Fick law [29]. Such a kind of behavior was also reported by Hietala et al. [26], attributing this result to a good dispersion of the reinforcement in the matrix, the high crystallinity of CNF, and the establishment of fibrous networks that prevent moisture penetration.

The second observation that must be noted is the lower water uptake capacity of composites compared with that of the plain TPS starch matrix (STO formulation). A similar behavior has been previously reported by Ghanbari et al. [30], attributed to the low resistance of starch to aqueous media. This result can be easily observed in the magnification of each profile, included in each trend in Figure 3. Again, this behaviour is related with the optimal distribution of fibers inside the material, allowing a good chemical interaction by hydrogen bonding between TPS and *CNF*_BEP_, decreasing the capacity for water diffusion through the material. When the water uptake assay at 7 °C/50% RH (Figure 3a) is compared with that at 23 °C/50% RH (Figure 3b), a slight decrease in water uptake of all the samples was obtained. The decrease in the stabilization temperature of the samples, keeping the humidity of the external medium constant, induced an increased water uptake capacity. This effect involved a decrease in the mechanical strength of the material, enhanced the elongation at break, and decreased the stiffness. This is the common plastifying result derived from the incorporation of small molecules of water inside the material. Moreover, increasing humidity in the external medium increased the incorporation of water content into the material, reaching values in the range of 20% to 25% *w*/*w*, when the relative humidity was set at 75%.

Other authors accept that this decrease in water uptake capacity can be explained by the establishment of hydrophobic interactions. Alves et al. [31] observed that, when microcrystalline cellulose (MCC) is regenerated from an aqueous dispersion of tetrabutylammonium hydroxide (TBAH) (40% *w*/*w* of TBAH in water), a low crystalline soft film of cellulose is obtained, opposite to the needle-shaped crystals obtained when MCC is regenerated from an aqueous solution of NaOH. A low crystalline cellulose gives rise to a material with lower capacity for hydrogen bonding, responsible for its crystallinity, and consequently lower capacity to interact with water [32].

### 3.3. Mechanical Characterization

**Figure 4:**
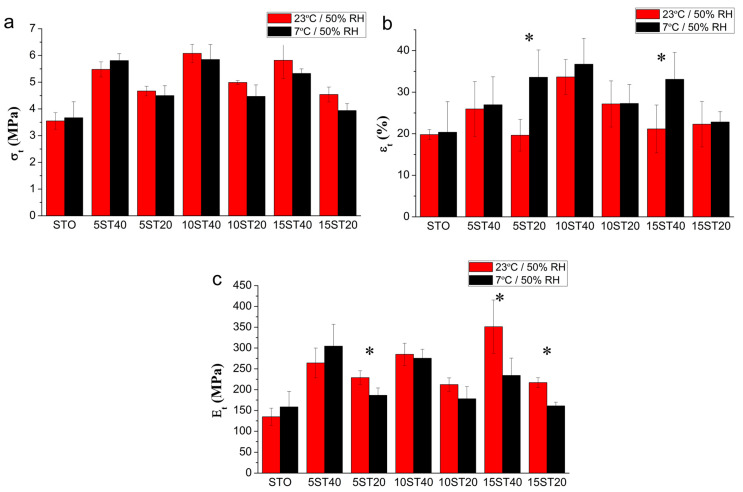
(**a**) Tensile strength (σt), (**b**) elongation at break (εt), and (**c**) Young’s modulus (Et) of starch composite films reinforced with *CNF*_BEP_. * This formulation shows statistical differences in the property at different temperatures.

Tensile strength (σ_t_), elongation at break (ε_t_), and Young’s modulus (E_t_) were determined according to ASTM D882 standard specifications and the results are summarized in Figure 4. Prior to the mechanical testing of the materials, the samples were stored under two different environmental conditions: 7 °C/50% RH and 23 °C/50% RH, simulating two different environmental conditions to which the material can be subjected during deployment (food contact).

The first observation related to mechanical characterization reveals a slight decrease of ultimate tensile strength regarding temperature, although from a statistical point of view, such a decrease is not significant. No significant differences in Young’s modulus, regarding temperature, were obtained either, with the exception of formulations 5ST20 and 15ST40, which show a statistical increase and corresponding decrease in elongation. This stiffening related to the decrease of temperature can be related to a lower mobility of the polymer chains owing to a lower temperature. The statistical differences of these formulations as well as those observed for 15ST20 can also be attributed to the high water sensibility of the components of the composite film during the mechanical characterisation owing to no chamber with controlled moisture/temperature being used. This result can be related with a different water content, and is expected to be the most influential parameter on the mechanical properties. As mentioned in Section 3.2, there are differences in water uptake observed at 7 °C and 23 °C, at constant relative humidity, but they are not enough to induce a different mechanical performance. There is a general overlapping of the averages of each property considering their standard deviation, denoting no significant differences (Figure 4).

However, the incorporation of *CNF*_BEP_ to the thermoplastic starch, plasticized with glycerol and water, revealed an important improvement in all mechanical properties. The percentage of reinforcement was also a parameter to be considered. The addition of 0.18% *w*/*w* of nanofibers represented an increase of the tensile strengths in the range from 7.3% to 22.6%, depending on the oxidation yield of the fibers, compared with the plain matrix. Otherwise, an improvement in σ_t_ from 45.3% to 58.3% was promoted by the incorporation of 0.36% *w*/*w* of *CNF*_BEP_, denoting the strengthening capabilities of CNF (Figure 4a). The fiber oxidation was also an important parameter, observing that the 10 mmol of NaClO treated reinforcement obtained the highest tensile strength enhancement. Altogether, the formulation that achieved the maximum value of σ_t_ was reinforced with 0.36% *w*/*w* of *CNF*_BEP_ fibers oxidized with 10 mmol NaClO. There are two main effects related to the oxidation degree: one positive, related to the increase in fibrillation because of the introduction of -COOH groups that induce repulsion between them forcing fibrillation; on the other hand, there is a negative effect related with the decrease in the molecular weight of the reinforcing agent because of the oxidation process. The characterisation of the -COOH content and the degree of polymerisation of *CNF*_BEP_ after TEMPO-induced oxidation was previously published by Serra et al. [33]. Carboxylic content was increased from 40 to 1392 μeq·g/g by the oxidation of 15 mmol of NaClO. This oxidation also reduced the polymerisation degree of cellulose from 1054 to 197. In terms of mechanical strength, equilibrium between the positive and negative effects is reached at a 10 mmols oxidation level. The effect of oxidation degree of the fibers on Young’s modulus does not show a clear trend.

The elongation at break was also improved by increasing the CNF contents (Figure 4b). This result is opposite to the main trend of composites reinforced with reinforcing agents at micro scale [34,35]. For the materials developed in this work, a good dispersion of the CNF in the final nanocomposite was assumed, giving the chemical compatibility and properties obtained. TEM results were attempted, but were not able to unambiguously discern the filler from the matrix. However, an indication for good dispersion is suggested by the fact that similar results, in terms of properties, were obtained by Martinez-Sanz et al. [28] in PLA reinforced with highly dispersed bacterial cellulose nanocrystals obtained by electrospinning. In our case, the good dispersion of the fibers is related to the homogenization process of the fibers, together with the plasticizer that avoided the formation of agglomerates.

Finally, Young’s modulus was increased by the addition of *CNF*_BEP_, showing again a dependence on the amount of reinforcement added to the composite (Figure 4c). However, the general trend of this parameter, regarding the external environmental conditions, is summarized as a decrease with decreasing temperature. Only the formulation 5ST40 showed an increase in Young’s modulus with decreasing temperature. This phenomenon was attributed to a plasticizing effect derived from the incorporation of a higher content of water at a lower temperature, and the results of the corresponding assay are shown and discussed as follows.

### 3.4. Hydrolytic Susceptibility in Enzymatic Media

**Figure 5:**
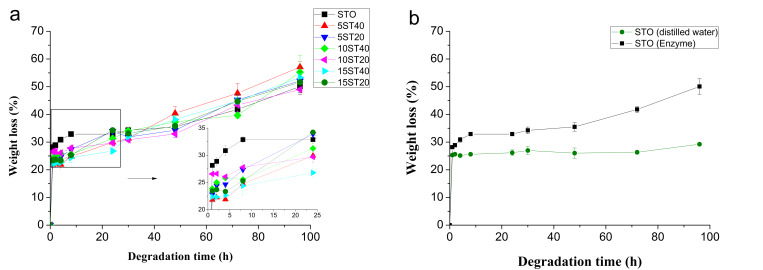
(**a**) Hydrolytic profiles of composite films based on thermoplastic starch as polymer matrix and CNF as reinforcement. (**b**) Comparison between STO behavior with and without enzymatic agents (distilled water).

This experiment was performed as an in vitro model to determine the effect of CNF on the hydrolytic susceptibility of starch. This property is important to confirm the biodegradable characteristic of the material. Starch has a very high hydrolytic susceptibility in the presence of enzymes such as α-amylases [36]. In this sense, the acquisition of hydrolytic profiles that can give information about this process is extremely dependent on the concentration of the enzyme in the external medium. So, the optimal enzyme concentration was adjusted to a hydrolysis time close to 4–5 days, leading to a concentration of α-amylase by 6 × 10^−2^ KNU/L (KNU = Kilo-Novo-Units α-amylase—the amount of enzyme that breaks down 5.26 g starch dry substance (Merck Amylum soluble No. 9947275 or equivalent) per hour). The results of the enzymatic degradation are summarized in Figure 5.

Hydrolysis and water uptake of the STO formulation (plain thermoplastic starch) followed a similar trend, showing a higher weight loss of this formulation during the first 35 h than the formulations reinforced with CNF (Figure 5a). This behavior is attributed to the high dispersions of *CNF*_BEP_ in the matrix and the establishment of hydrogen bonding interactions with the matrix. This interaction kept the structural integrity of the composite against penetration of water and enzymatic degradation.

The optimized enzyme concentration adjustment allows to visualize both stages that can be observed: (1) before 35 h of immersion and (2) after this time. The pseudo-plateau acquired after the first 1–2 h represents the leaching process of the plasticizers (water and glycerol). As mentioned above, the presence of the fibers kept the integrity of the systems reaching values of weight loss lower than that of STO. During this time period, the rate of hydrolysis was lower than the rate of leaching of the plasticizer, giving rise to this pseudo-plateau. Once the immersion time was increased, after 35 h, the slope of the trend was increased, again corresponding to the real enzymatic hydrolytic process. To confirm this hypothesis, another experiment was performed, where a sample of STO was submitted to two different aqueous media: (1) distilled water and (2) enzymatic medium, both at 50 °C, and the results are shown in Figure 5b. It is easy to observe that the sample submitted to distilled water has no slope change after 35 h, while that immersed in the enzymatic medium does show a change. Moreover, a higher weight loss in the material was observed before 35 h for the sample immersed in the enzymatic medium because of the competence between the plasticizer leaching process and enzymatic degradation.

### 3.5. Oxygen and Water Permeability

The barrier mechanism of nanoparticles in nanostructured reinforced composites is based on the hindering capacity that those nanoreinforcements can induce to the polymer matrix to prevent the transport of a gas through the thickness of the material. The presence of particles in the way of transport of the gas through the material increases the length and the time of gases to cross the material. This phenomenon has attracted huge interest in the fabrication of packaging elements to prevent the contact of aliments with air for a longer period of time, increasing the preservation of foods. So, the incorporation of nanostructured cellulose to starch could decrease the susceptibility of this polymer matrix to be permeated by gases that can damage the conservation and visual aspect of fresh foods. In this work, films of STO (plain thermoplastic starch), 10ST20 and 10ST40, reinforced with 0.18% and 0.36% *w*/*w* of *CNF*_BEP_, respectively, were evaluated for their water vapour and oxygen permeability. These two gases were chosen owing to their direct relationship with damage of fresh food during conservation.

The permeation values are gathered in Table 2. From the results, it appears that a loading of 0.18% *w*/*w* of nanofibers did not result in a measurable barrier effect, most likely derived from an inefficient tortuosity role of the scarce nanofiller. Although some differences in the transport of water vapour or oxygen were registered for 10ST20, no statistical differences were obtain as studied by one-way ANOVA statistical test. However, the incorporation of a higher loading (0.36% *w*/*w*) resulted in a clear gas permeability drop up to 56% for water vapour and 51% for oxygen. This result corroborates the mechanism of diffusion of the gas through the material, where the concentration of the nanoreinforcement is a key factor to induce barrier character. One-way ANOVA statistical test also demonstrates that values of diffusion of water vapour and oxygen dropped statistically through 10ST40 and are significantly lower compared with STO control. Owing to this result, the barrier characterisation suggests that the potential shelf-life extension of a package in which this nanofiller was incorporated as a barrier element could be more than double.

## 4. Conclusions

The incorporation of the reinforcement (CNF) to the plasticizer (glycerol), prior to nanofibrillation in a homogenizer, allowed obtaining a characteristic gel-like texture of CNF by the establishment of the corresponding hydrogen bonding. The incorporation of such gel as reinforcing agent to native starch powder gave rise to materials with a good compatibility, attributed to the dispersion improvement of the CNF in the polymer matrix. These materials showed improvement in mechanical properties, barrier character, and decrease in water uptake. An improvement in the mechanical properties (tensile strength, elongation at break, and Young’s modulus) suggested a better dispersion, assuming a good interaction between both components of the composite by hydrogen bonding. This bonding also allowed the material to obtain a lower water uptake capacity, compared with the plain polymer matrix, with the environmental conditions of 7 °C/23% RH being those with a lower rate of water diffusion. The incorporation of this reinforcement did not harm the hydrolytic susceptibility characteristic, understood as the degradation capacity of starch when the material was immersed in an enzymatic active medium.

From the study of the hydrolytic behaviour, the incorporation of 0.36 *w*/*w* of CNF produced a decrease in gas diffusion to the composite film, offering a double value in the food preservation time.

In conclusion, the methodology of pre-addition of CNF in the plasticizer, avoiding the incorporation of extra water quantities to the polymer matrix, opens a new way to prepare CNF reinforced composite materials based on hydrophilic matrices, by a different methodology to solvent-casting, diminishing the probability of obtaining aggregates of the reinforcing agent and improving the mechanical performance as well as the barrier character for food preservation materials.

## Figures and Tables

**Table 1:** Composition and nomenclature of thermoplastic starch films reinforced with *CNF*_BEP_ fibers. * NaClO added to oxidize bleached eucalyptus pulp (BEP); ϕ: *CNF*_BEP_ dispersed in glycerol (concentration of 1.0% *w*/*w*); ++ *CNF*_BEP_ included in the formulation; ‡ % of *CNF*_BEP_ in the composite film considering the feed composition of the whole composite.

Formulation	Oxidation * (mmol)	Starch (g)	Gly-*CNF*_BEP_^ϕ^ (g)	*CNF*_BEP_^++^ (g)	Glycerol (g)	H_2_O (g)	*CNF*_BEP_^‡^ (%)
ST0 (ref.)	---	60	---	---	40	12	---
5ST20	5	60	20	0.2	20	12	0.18
5ST40	5	60	40	0.4	---	12	0.36
10ST20	10	60	20	0.2	20	12	0.18
10ST40	10	60	40	0.4	---	12	0.36
15ST20	15	60	20	0.2	20	12	0.18
15ST40	15	60	40	0.4	---	12	0.36

**Table 2:** Values of water vapor permeation (WVP) and oxygen permeation (OP) of STO (reference), 10ST20 and 10ST40 formulations. Each material was characterised twice and both individual results are shown, referred as −1 and −2. ^a^: no significant differences regarding control STO; ^b^: significant differences regarding control.

Sample	WVP·10^13^ (Kg·m·Pa^−1^ s^−1^·m^−2^)	OP·10^19^·(m^3^·m·Pa^−1^·s^−1^·m^−2^)
STO-1	6.19 (0.72) ^a^	1.11 (0.06) ^a^
STO-2	5.90 (0.90) ^a^	1.23 (0.04) ^a^
10ST20-1	6.63 (0.85) ^a^	1.35 (0.13) ^a^
10ST20-2	5.65 (0.28) ^a^	1.16 (0.15) ^a^
10ST40-1	2.96 (0.71) ^b^	0.54 (0.01) ^b^
10ST40-2	2.72 (0.85) ^b^	0.60 (0.06) ^b^
